# Laryngeal Clear Cell Carcinoma: A Systematic Review

**DOI:** 10.1002/oto2.70157

**Published:** 2025-09-02

**Authors:** Gabriele Noreikaite, Savannah Nicks, Daniel Lofgren, Kerolos Shenouda, Olga Santiago Rivera

**Affiliations:** ^1^ Department of Otolaryngology–Head & Neck Surgery McLaren Oakland Hospital Pontiac Michigan USA; ^2^ Department of Otolaryngology–Head & Neck Surgery Vanderbilt University Nashville Tennessee USA

**Keywords:** head and neck cancer, laryngeal carcinoma, laryngeal clear cell carcinoma, non‐squamous cell carcinoma of the larynx

## Abstract

**Objective:**

Laryngeal clear cell carcinoma (LCCC) is an exceedingly rare tumor. Current literature on LCCC is limited to case reports with little comprehensive data available. This systematic review aims to analyze existing literature to better characterize LCCC and to identify trends in presentation, treatment, and survival.

**Data Sources:**

A literature search of PubMed, MedLine, and Embase was conducted.

**Review Methods:**

A systematic review of LCCC cases from 1976 to 2024 was performed. Data extraction followed PRISMA guidelines. Included studies were those describing adult or pediatric patients pathologically diagnosed with LCCC. Excluded studies were those describing nonclear cell pathology, nonlaryngeal location, or nonprimary clear cell carcinoma.

**Results:**

In total, 7 studies (n = 9 patients) were included in the analysis. Males (77.7%) were more commonly affected, with an average patient age of 56.5 years. The supraglottis was the most frequently involved subsite (66.6%). Most patients presented with advanced locoregional disease. Surgery alone was the most common treatment (66.6%), followed by chemoradiation (22.2%) and surgery with adjuvant chemoradiation (11.1%). Disease recurrence occurred in 55.5% of cases. Four patients (44.4%) died due to the disease, with an average survival of 9.3 months.

**Conclusion:**

LCCC is a rare tumor often presenting as a supraglottic mass with cervical metastasis. Patients underwent various treatments with surgery, chemoradiation, or a combination of both. Reported survival was generally poor, emphasizing the aggressive nature of this disease.

Primary clear cell carcinoma (CCC) is a rare type of cancer which mainly develops within the renal system, lungs, liver, pancreas, and female reproductive organs. The name arises from the classic histologic appearance of voluminous cells with large, clear cytoplasm arranged in nests. Based on histological staining, clear cell carcinoma can further be divided into squamous or adenocarcinoma type, both of which are considered poorly differentiated.^1^

In the head and neck, primary clear cell carcinoma has been described as a minor salivary gland tumor that can affect the entire aerodigestive tract including the nasal cavity and paranasal sinuses, oral cavity, pharynx, and larynx.^2^ In 2020, Mukdad et al conducted a SEER‐based retrospective cohort study of head and neck clear cell adenocarcinoma and reported the oral cavity (35%) and salivary glands (34%) as the most common sites for this tumor. Laryngeal involvement is exceedingly rare, making up only 0.6% of head and neck CCC cases.

CCC of the larynx was first described in 1976 and included in the World Health Organization (WHO) classification in 1978.^2^, ^3^ The exact etiology of these tumors is unknown. However, they tend to arise from submucosal minor salivary glands within the larynx. The largest number of minor salivary glands are in the subglottis followed by the supraglottis in the false true vocal folds, aryepiglottic folds, and the epiglottis. In the glottis, they are located within the subglottic surface of the anterior commissure. As most minor salivary gland tumors, CCC of the larynx is malignant in nature and must be differentiated from other minor salivary gland etiologies such as adenosquamous carcinoma, mucoepidermoid carcinoma, adenoid cystic carcinoma, and acinic cell adenocarcinoma.^4^, ^5^

The current literature on CCC of the larynx is composed of individual observational studies with little comprehensive data available on epidemiology, treatment, and survival. The goal of this qualitative systematic review is to examine the available literature on laryngeal CCC to better characterize the disease and determine trends in presentation, treatment, and patient outcomes.

Understanding these patterns is essential for otolaryngologists treating future patients, as it may provide insight into optimal management strategies, including the role of surgical versus nonsurgical treatment modalities and the potential benefits of early diagnosis.

## Methods

**Figure 1:**
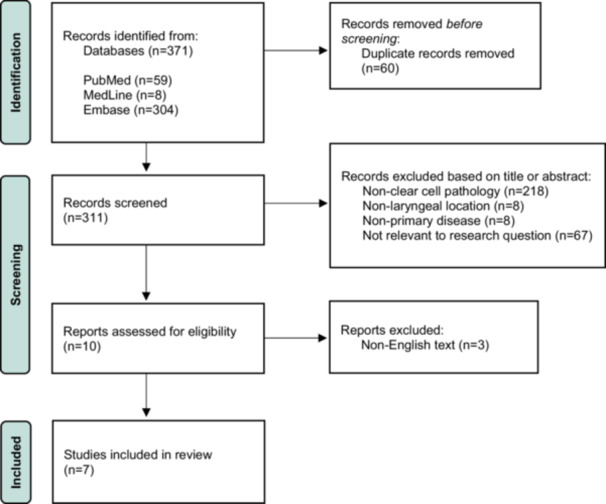
Article selection process according to preferred reporting items for systematic reviews and meta‐analysis guidelines.

This study was designed as a systematic review of the literature of laryngeal clear cell carcinoma (LCCC). The article selection process used can be seen in Figure 1, which follows the Preferred Reporting Items for Systematic Reviews and Meta‐Analyses (PRISMA) guidelines.

Inclusion criteria included any study type from 1976 to 2024 containing adult and/or pediatric patients who were pathologically diagnosed with LCCC. English language or English translated papers were included, and the authors were required to have full access to abstract and manuscript.

Exclusion criteria involved studies that were determined by the authors to be letters to the editor, opinion pieces, duplicated studies, unavailable full texts, non‐English written texts, non‐laryngeal location, nonclear cell pathology, nonprimary laryngeal CCC (eg, renal CCC metastasizing to the larynx), and texts not relevant to the research question.

The following online databases were used to identify appropriate studies that met our inclusion and exclusion criteria: PubMed, MedLine, and Embase. The databases were last accessed March 2024. The following MesH search terms were used to further identify articles: “clear cell carcinoma larynx” OR “laryngeal clear cell carcinoma” OR “supraglottic clear cell carcinoma” OR “supraglottis clear cell carcinoma” OR “glottic clear cell carcinoma” OR “glottis clear cell carcinoma” OR “subglottic clear cell carcinoma” OR “subglottis clear cell carcinoma” NOT “squamous.”

All abstracts for the studies were screened and reviewed by the lead authors (GN, SN) to determine if the studies met the inclusion or exclusion criteria above. Any dispute on inclusion or exclusion was independently reviewed by the third author (DL). For data collection, all studies were reviewed independently by the authors. The author team independently reviewed each paper to determine the significance of the topic and remove any duplicates.

Case reports and case series are inherently biased; however, standardized tools have been developed to assess their quality. The JBI Critical Appraisal Checklist for Case Reports and Case Series was used for article quality appraisal and assessment of bias.^6^, ^7^ Studies were rated as having low, moderate, or high risk of bias. Lead authors (GN, SN) independently appraised each article for quality and risk of bias. Any disputes were reviewed by the third author (DL). All authors followed the above criteria.

Given the descriptive nature of this review, the authors used descriptive statistics to report demographics and clinical findings. Means and standard deviations were used for continuous variables such as age. Frequencies and percentages were used for dichotomous variables such as symptoms and physical exam findings.

## Results

The initial search produced 371 titles, which were imported into Zotero for duplicate removal. After duplicates were excluded, 311 titles and abstracts were screened using the inclusion and exclusion criteria described in the Methods section. Ten full‐text articles were reviewed, and 3 were excluded due to non‐English text without available translations. Ultimately, 7 articles met criteria for qualitative analysis. A summary of these articles is shown in Table 1. Of the 7 included studies, 6 (86%) were case reports and 1 (14%) was a case series including 3 patients.

**Table 1:** Studies on Laryngeal Clear Cell Carcinoma

Study	Study design	N (*p*)	Quality rating
Pesavento (1980)	Case series	3	4
Testa (2005)	Case report	1	5
Seo (1980)	Case report	1	5
Halder (2020)	Case report	1	5
Nayak (2000)	Case report	1	5
Palma and Blandamura (1989)	Case report	1	5
Hoff (2011)	Case report	1	5

Using the JBI Critical Appraisal Checklist for Case Reports and Case Series, 5 studies (71%) were determined to be low risk for bias, 1 study (14%) was moderate risk, and 1 study (14%) was high risk.^6^, ^7^

A total of 9 patients were included across the selected studies. Demographic and clinical characteristics are summarized in Table 2. Most patients were male (n = 7, 78%) with a mean age of 56.5 years (range: 15‐76). Race and social history, including tobacco and alcohol use, were not consistently reported and therefore were not included in the analysis.

**Table 2:** Demographics of Patients with Laryngeal Clear Cell Carcinoma

Characteristic	No. (% of total, N = 9)
Sex	
Male	7 (77.7)
Female	2 (22.2)
Age, y	
0‐29	1 (11.1)
30‐49	2 (22.2)
50‐69	3 (33.3)
70‐89	3 (33.3)
Mean (SD)	56.5 (–)
Range	15‐76
Presenting symptoms	
Hoarseness	4 (44.4)
Dysphagia	2 (22.2)
Cough	1 (11.1)
Dyspnea	1 (11.1)
Hemoptysis	1 (11.1)
Physical examination findings	
Bilateral lymphadenopathy	3 (33.3)
Unilateral neck mass	1 (11.1)
No pertinent findings	5 (55.5)
Flexible fiberoptic laryngoscopy findings	
Ulcerative laryngeal lesion	3 (33.3)
Fungating/exophytic laryngeal lesion	6 (66.6)
Lesion subsite	
Supraglottis	6 (66.6)
Glottis	2 (22.2)
Subglottis	1 (11.1)

The most common presenting symptom was hoarseness followed by dysphagia, cough, dyspnea, and hemoptysis. On physical examination, cervical lymphadenopathy was commonly noted; however, many patients had no pertinent findings. Flexible laryngoscopy revealed a fungating or ulcerative laryngeal mass which was most frequently involved the supraglottic laryngeal subsite.

Staging, treatment, and survival data is shown in Table 3. The most common stage at presentation was advanced locoregional disease with extension to the cervical lymph nodes. Three patients had localized disease without nodal involvement. No patients presented with distant metastasis.

**Table 3:** Staging, Treatment, and Survival Data of Patients with LCCC

Patient	Site	Stage	Treatment	Recurrence/site	Disease‐free interval, mo	Salvage therapy	Survival, mo	Death due to disease
1	Supraglottis	pT3N2c	TL, BND	Yes/hypopharynx	14	No	18	Yes
2	Supraglottis	pT4N2c	Supraglottic laryngectomy, BND	Yes/hypopharynx	2	No	3	Yes
3	Supraglottis	pT2N2c	TL, BND	Yes/BOT	5	Radiation	6	Yes
4	Supraglottis	pT2	Supraglottic laryngectomy, BND	No	NR	No	28+	No
5	Supraglottis	pT4N2c	TL, BND, adjuvant chemoradiation	No	NR	No	18+	No
6	Supraglottis	cT3N2cM0	Chemoradiation	No	NR	No	6+	No
7	Glottis	cT2N0	Frontolateral laryngectomy	No	NR	No	24+	No
8	Glottis	pT4N2b	TL, R ND	Yes/cervical lymph node	6	No	10	Yes
9	Subglottis	cT1	Radiation	Yes/trachea	6	TL, partial tracheal resection	7	No^a^

Abbreviations: BND, bilateral neck dissection; BOT, base of tongue; NR, no recurrence; R ND, right neck dissection; TL, total laryngectomy.

^a^ Death due to cardiovascular incident.

Surgical treatment alone was the most common treatment modality (n = 6, 67%). Surgical approaches included total or partial laryngectomy, with or without neck dissection. Two patients (22%) were treated nonsurgically—1 with chemoradiation and 1 with radiation alone. One patient (11%) was treated with surgery (total laryngectomy and bilateral neck dissection) followed by adjuvant chemoradiation.

Five patients (56%) experienced disease recurrence after treatment. Of those patients, 2 (40%) underwent salvage therapy. None of the patients with disease recurrence survived regardless of salvage therapy. Overall, 4 patients (44%) died secondary to disease progression.

## Discussion

Clear cell carcinoma of the head and neck is a rare entity most commonly affecting the oral cavity followed by the major salivary glands. Laryngeal involvement has been reported in only 0.6% of head and neck CCC cases.^2^, ^8^, ^9^, ^10^ Succinct data is not currently available on epidemiology and treatment of the disease making it difficult for otolaryngologists, oncologists, and other healthcare providers to adequately manage the patient with laryngeal CCC. To our knowledge, this is the first reported systematic review of laryngeal CCC.

As noted in multiple studies, CCC of the head and neck appears to affect females disproportionately to males (54%).^2^, ^10^ In contrast, our study focusing solely on laryngeal CCC showed an overwhelming male predominance (78%). Mean age of patients diagnosed with LCCC is 56.5 which is consistent with the mean age of patients with H&N CCC (61) as well as patients with malignant minor salivary gland tumors of the larynx (54).^2^, ^10^, ^11^, ^12^, ^13^, ^14^, ^15^ Information on social history such as tobacco and alcohol use was not included in the review, because a majority of the studies did not report it. However, malignant minor salivary gland tumors of the larynx are not strongly associated with tobacco and alcohol use.^8^

The most common presentation of laryngeal CCC is persistent hoarseness followed by dysphagia. Approximately half the patients in the reported cases additionally presented with either unilateral or bilateral cervical lymphadenopathy consistent with local metastasis. Because these tumors typically present in a subepithelial fashion, signs and symptoms of the disease do not become apparent until the tumor has significantly progressed. With increasing tumor size, the risk of cervical lymph node metastasis increases.^8^, ^9^

The anatomical distribution of the submucosal glands in the larynx is greatest in the subglottis.^2^, ^4^, ^8^ Based on the distribution of the submucosal glands, the majority of laryngeal CCC should arise from the subglottis; however, our study shows that 67% of patients with laryngeal CCC presented with tumors of the supraglottis. The discrepancy in findings could be secondary to the small sample size of the study or a specific predominance of CCC to the supraglottis despite it being a minor salivary gland tumor.

Of the patients with supraglottic disease, all presented with at least T2 disease based on the TNM staging system for laryngeal carcinomas. Five of these patients (83%) also had nodal disease which was noted either on physical exam or intraoperatively. Distant metastatic disease was not reported in any of the patients. Three patients (60%) with nodal disease underwent surgical treatment, and all patients experienced disease recurrence with subsequent death due to disease. The mean months survived was 9.3. One patient (20%) with nodal disease underwent treatment with chemoradiation and 1 (20%) underwent treatment with combination chemoradiation and surgery. Both patients survived 18+ months without reported recurrence or death due to disease. Lastly, 1 patient with supraglottic disease without any nodal metastases underwent treatment with surgery and survived 28+ months without reported recurrence or death secondary to disease.

A similar trend is seen with glottic disease. Of the 2 patients, both presented with at least T2 disease. One presented with nodal disease and the other did not. Both patients underwent treatment with surgery alone, and the patient without nodal disease survived 24+ months without reported recurrence or death due to disease. The patient with nodal disease experienced local recurrence after surgical treatment and died due to disease 10 months later.

Treatment approaches varied among reported cases. Patients with nodal disease were treated with surgery, chemoradiation, or a combination of both. Some reports showed longer survival and no recurrence in those receiving chemoradiation. Patients without nodal disease treated with surgery alone appeared to have prolonged survival without recurrence based off the limited reported cases. While definitive conclusions cannot be validated based on this descriptive review, these patterns seem to differ from treatment recommendations of other minor salivary gland tumors. A systematic review on the treatment and outcomes of minor salivary gland cancers of the larynx and trachea by Montenegro et al showed that surgery with or without adjuvant chemoradiation was the preferred treatment for laryngeal minor salivary gland tumors. Chemoradiation alone was reserved for patients with distant metastasis. A retrospective study by Ganly et al reported similar recommendations for surgical treatment of malignant minor salivary gland tumors of the larynx due to the relative radioresistant nature of these tumors.^4^, ^9^

The presented systematic review has some limitations. Due to the rarity of laryngeal CCC, available studies were limited to case studies and series which provide mainly qualitative data. The sample size of the combined studies was only 9 patients which inherently limits the statistical power and generalizability of the findings. Additionally, the retrospective and descriptive nature of the included studies increases the potential for selection and reporting bias. Of the available reports, many of them were incomplete in disease staging and survival data. Variations in staging terminology, treatment protocols, and outcome definitions complicated direct comparisons. Lastly, most of the included studies were published prior to 2000 and may not reflect advancements in surgery, radiation, and chemotherapeutic agents that have since become standard in head and neck cancer treatment.

Despite these limitations, this is the first study to systematically review published cases of laryngeal CCC and report trends in patient demographics, disease treatment, and survival. The authors hope this study will aid otolaryngologists, oncologists, and multidisciplinary teams in managing this rare malignancy, and will serve as a foundation for future investigation.

## Conclusion

Laryngeal clear cell carcinoma is an extremely rare malignancy, making up only 0.6% of CCC in the head and neck. Nonetheless, it is an aggressive form of cancer which often presents with locoregional metastasis. In large part due to the rarity of the disease, there is currently insufficient data available to guide clinical decision making. This review serves as a foundation for otolaryngologists that may aid in the management of future cases and contribute to the literature of this rare malignancy.

## Author Contributions

**Gabriele Noreikaite**: conceived the design of the study, performed data collection, interpretation, and drafted the manuscript; **Savannah Nicks**: performed data collection and provided revisions to the content of the manuscript; **Daniel Lofgren**: provided revisions to the content of the manuscript; **Kerolos Shenouda**: principal investigator; **Olga Santiago**: performed data interpretation.

## Disclosures

### Competing interests

None. 

### Funding source

None.
